# Efficacy and safety of tart cherry supplementary citrate mixture on gout patients: a prospective, randomized, controlled study

**DOI:** 10.1186/s13075-023-03152-1

**Published:** 2023-09-07

**Authors:** Can Wang, Wenyan Sun, Nicola Dalbeth, Zhongjun Wang, Xuefeng Wang, Xiaopeng Ji, Xiaomei Xue, Lin Han, Lingling Cui, Xinde Li, Zhen Liu, Aichang Ji, Yuwei He, Mingshu Sun, Changgui Li

**Affiliations:** 1 Shandong Provincial Key Laboratory of Metabolic Diseases and Qingdao Key Laboratory of Gout, the Affiliated Hospital of Qingdao University, Qingdao, China; 2 Institute of Metabolic Diseases, Qingdao University, Qingdao, China; 3 Shandong Provincial Clinical Research Center for Immune Diseases and Gout, Qingdao, China; 4 Department of Medicine, University of Auckland, Auckland, New Zealand; 5 Department of Clinical Laboratory, the Affiliated Hospital of Qingdao University, Qingdao, China; 6 Department of Rheumatology, the Affiliated Hospital of Qingdao University, Qingdao, China

**Keywords:** Gout, Urine alkalization, Sodium bicarbonate, Citrate, Tart cherry

## Abstract

**Background:**

Low urine pH, which may be mediated by metabolic syndrome (MetS), is common in gout. Tart cherries are shown to improve MetS symptoms and possess anti-inflammatory properties. However, the efficacy of tart cherry supplements on urine pH has yet to be studied.

**Objectives:**

This study aimed to investigate the efficacy and safety of tart cherry supplementary citrate (TaCCi) mixture on urine pH, serum urate (sUA), C-reactive protein (CRP), and gout flares in gout patients initiating urate-lowering therapy (ULT), in comparison to citrate mixture and sodium bicarbonate.

**Methods:**

A prospective, randomized (1:1:1), open-label, parallel-controlled trial was conducted among 282 men with gout and fasting urine pH ≤ 6, who were initiating ULT with febuxostat (initially 20 mg daily, escalating to 40 mg daily if serum urate ≥ 360 μmol/L). Participants were randomized to groups taking either sodium bicarbonate, citrate mixture, or TaCCi mixture. All participants were followed every 4 weeks until week 12. Urine pH and sUA were co-primary outcomes, with various biochemical and clinical secondary endpoints.

**Results:**

Urine pH increased to a similar extent in all three groups. SUA levels declined in all three groups as well, with no significant differences observed between the groups. At week 12, the TaCCi mixture group exhibited a greater reduction in the urine albumin/creatinine ratio (UACR) compared to the other two groups (*p* < 0.05). Participants taking TaCCi mixture or citrate mixture experienced fewer gout flares than those in the sodium bicarbonate group over the study period (*p* < 0.05). Additionally, the TaCCi mixture group had a lower CRP level at week 12 relative to the other two groups (*p* < 0.01). Adverse events were similar across all three groups.

**Conclusion:**

The TaCCi mixture had similar efficacy and safety on urine alkalization and sUA-lowering as the citrate mixture and sodium bicarbonate in patients with gout. However, the TaCCi mixture resulted in greater improvements in UACR and CRP, which suggests that tart cherry supplements may provide additional benefits for renal protection and reduce inflammation in gout, particularly when starting ULT.

**Trial registration:**

This project was registered in ChiCTR (www.chictr.org.cn), with the registration number: ChiCTR2100050749.

**Supplementary Information:**

The online version contains supplementary material available at 10.1186/s13075-023-03152-1.

## Background

Gout is the most common inflammatory arthritis worldwide, with a high prevalence of comorbidities including metabolic syndrome (MetS) [1–3]. In addition to elevated serum and urinary urate levels, low urine pH is very common among patients with gout and primary hyperuricemia [4, 5]. A previous cross-sectional study among 3565 primary gout patients in our institution found that 46.5% had aciduria (pH < 5.5), and low urine pH was independently linked to diverse kidney injuries, including chronic kidney disease (CKD), nephrolithiasis, renal cyst, traces of hematuria and proteinuria [6]. The low urine pH in gout may be mediated by MetS, which is associated with impaired ammoniagenesis and citrate excretion of the renal proximal tubules [7, 8].

There is growing interest in the role of tart cherries as complementary supplements for gout management [9]. Tart cherries contain high levels of anthocyanins, which have potent anti-inflammatory and anti-oxidative properties [10]. Previous studies suggest tart cherry supplements may provide benefits for MetS. In one animal study, a tart cherry-enriched diet decreased total cholesterol (TC) and low-density lipoprotein cholesterol (LDL-C) levels, while increasing the high-density lipoprotein cholesterol (HDL-C) concentration [11]. In humans, cherry juice supplementation was shown to attenuate systolic blood pressure [12] and improve 24-h blood pressure, fasting blood glucose (FBG) levels, TC, LDL-C, and TC/HDL-C ratio [13]. Furthermore, some studies found that cherries and cherry products lowered serum urate (sUA) levels in healthy or obese individuals [14, 15], though this effect has not been consistently observed[16]. Additionally, cherries and cherry products may benefit gout patients, with research finding a 35% lower risk of gout flares [17].

Urine alkalinization may help limit urolithiasis and enhance the effectiveness of urate-lowering therapy (ULT) [18]. However, recommendations regarding urine alkalization in gout management guidelines have been inconsistent due to insufficient evidence. Some guidelines have suggested considering urine alkalization in limited clinical settings [19, 20], while the 2020 American College of Rheumatology (ACR) gout management guideline recommended against urine alkalization [21]. Maintaining the sUA levels below target through ULT is the key strategy for gout management. Xanthine oxidase inhibitors (XOI), such as allopurinol and febuxostat, are strongly recommended as the first-line ULT drugs [21, 22]. XOI treatment decreases the urinary urate concentration and may reduce the formation of uric acid crystals and stones, but this may not necessarily apply to patients with aciduria.

We have previously reported that a citrate mixture had comparable efficacy to sodium bicarbonate on urine alkalization in patients with gout and was superior in reducing hematuria as well as gout flares [23]. However, the 24-h urinary albumin/creatinine ratio (UACR) was not measured in that study, nor were any improvements found in MetS components [23]. Here we report the results of a clinical trial testing the efficacy and safety of tart cherry supplementary citrate (TaCCi) mixture in gout patients with XOI febuxostat initiation and acidic urine, compared to a citrate mixture and sodium bicarbonate. The study hypothesized that the tart cherry supplement would have an additional beneficial effect on urine pH and sUA through improvements in MetS.

## Methods

### Study design and participants

This was an open-labeled, prospective, randomized, parallel controlled trial, conducted between September 2021 and June 2022 at the Gout Clinic of the Affiliated Hospital of Qingdao University. A totally of 354 participants were recruited. The study was conducted in accordance with the Declaration of Helsinki and Good Clinical Practice, and the protocol was reviewed and approved by the Ethics Committee of the Affiliated Hospital of Qingdao University.

The trial was registered in ChiCTR (www.chictr.org.cn), with the registration number ChiCTR2100050749. Eligible participants were male, aged between 18 and 70 years, met the 2015 ACR/EULAR classification criteria for gout, and were about to initiate ULT [24], with fasting urine pH ≤ 6. Due to the low prevalence of gout in women [25] and to minimize the sex confounding influences, only male participants were included in this study. Exclusion criteria included: on ULT or experienced gout flare within 14 days before recruitment; estimated glomerular filtration rate (eGFR) < 60 ml/min/1.73 m^2^; sUA < 420 μmol/L or > 600 μmol/L; transaminase > two folds of the upper limit of normal (ULN); taking other drugs affecting sUA and/or urine pH; allergic to any drugs or ingredients involved in this trial; secondary gout and secondary hypertension. All participants gave their written informed consents before screening for eligibility. Eligible participants were 1:1:1 randomly assigned to the sodium bicarbonate, citrate mixture, or TaCCi mixture group.

### Procedures

All participants were required to undergo a 14-day washout period to withdraw any drug with urate-lowering potential and follow a low-purine diet before baseline data was collected. The random number creator was used to generate a randomization list for those eligible participants, who were 1:1:1 randomly assigned to the sodium bicarbonate group (sodium bicarbonate 1 g, three times a day), the citrate mixture group (citrate mixture: citric acid 50%, sodium citrate 10%, potassium citrate 10%, sodium carbonate 20% and excipient 10%, 3.5 g, twice a day) or the TaCCi mixture group (TaCCi mixture: tart cherry powder 25%, citric acid 30%, sodium citrate 2%, potassium citrate 2%, sodium carbonate 30% and excipient 11%, 3.5 g, twice a day). All participants started ULT with febuxostat (initially 20 mg daily, escalating to 40 mg daily if sUA ≥ 360 μmol/L at first follow-up). Colchicine or other gout flare prophylaxis drugs were not eligible in the trial protocol, while etoricoxib 120 mg once a day for 3–5 days would be prescribed for patients who experienced gout flare during the study. Participants were followed every 4 weeks at a face-to-face visit until week 12.

Demographic and medical history data were acquired at the baseline visit, including age, nephrolithiasis, tophi, smoking, and drinking history. Blood and urine parameters including sUA, triglycerides (TG), TC, FBG, homeostasis model assessment of insulin resistance (HOMA-IR) [26], transaminase, blood urea nitrogen (BUN), serum creatinine (CREA), eGFR, urine pH, urine protein and hemoglobinuria as well as blood pressure (BP) and body mass index (BMI) were obtained at every visit. Other parameters were tested at baseline and week 12 including dual-energy CT (DECT) of affected joints, kidney ultrasonography, 24-h UACR, C-reactive protein (CRP), serum potassium (K^+^), serum sodium (Na^+^) and serum chloride (Cl^−^). Proteinuria and hemoglobinuria were reported as positive when the urinalysis urine protein or hemoglobin reading of ≥  + / − in the urine sample. The pH of the fresh fasting urine samples was determined using a pH electrode (FE28-STANDARD, METTLER Toledo Company, Zurich, Switzerland); Kidney ultrasonography examination was performed using an ALOKA 70 machine (HITACHI, Tokyo, Japan); All symptomatic joints were scanned on a dual x-ray tube 128-detector-row scanner (Somatom Definition Flash, Siemens Healthcare, Forchheim, Germany), with tube A 140kVp/55mAs and tube B 80kVp/255mAs, acquisition at 128 × 0.6 mm, reconstruction at 0.6 mm. Urate volumes were automatically calculated by DECT Syngo via the Gout program (Siemens Healthcare, Germany).

Adverse events were monitored and managed during the study period. Gout flare was defined as a patient-reported flare with pain VAS score > 3 of 0–10 scale [27]. Newly-onset hypertension, which was defined as a systolic blood pressure ≥ 140 mmHg and/or a diastolic blood pressure ≥ 90 mmHg or under antihypertensive treatment or diagnosed by a physician during the follow-up [28]. Polyene phosphatidylcholine would be administered if the transaminase increased to ≥ 2-folds of the ULN, and etoricoxib 120 mg once a day for 3–5 days would be administrated for gout flares.

### Outcomes

The primary outcomes were changes in urine pH and sUA level over 12 weeks. Gout-related outcomes included changes of CRP level, gout flares and changes in DECT-detected monosodium urate (MSU) volume. The renal and metabolic outcomes included changes in eGFR, UACR, and MetS components (including BP, BMI, FBG, HOMA-IR, and blood lipids). Adverse events included changes in serum electrolyte level, newly-onset hemoglobinuria, newly-onset nephrolithiasis/renal cyst examined by kidney ultrasonography, newly-onset hypertension, skin allergy, transaminase elevation, and other adverse events that might lead to treatment interruption or hospitalization.

### Sample size

Sampling was based on the joint primary outcomes, urine pH, and sUA. In a separate pilot study conducted in our center, 60 participants were included under the same inclusion/exclusion criteria, the mean change of pH mean ± standard derivation (S.D) was 0.40 ± 0.517, 0.38 ± 0.371, and 0.64 ± 0.478 in the sodium bicarbonate group, citrate mixture group and TaCCi mixture group, respectively. To detect a 0.232 difference in urine pH between sodium bicarbonate and TaCCi mixture, 75 patients in each group provided > 80% power at a significance level of 0.05. In the pilot study, the mean change of sUA (mean ± S.D) was 134 ± 93.5 μmol/L, 137 ± 80.6 μmol/L, and 175 ± 76.1 μmol/L in the sodium bicarbonate group, citrate mixture group, and TaCCi mixture group, respectively. To detect the 38.4 μmol/L difference in sUA between the citrate mixture group and TaCCi mixture group, 69 patients in each group were required. Based on the urine pH and sUA data, 75 participants in each group would provide > 80% power at a significance level of 0.05. Considering a drop-out rate of 20%, at least 281 participants were needed.

### Statistical analysis

Demographic and clinical features were displayed using standard descriptive statistics including mean ± S.D, median (interquartile range), or number (percentage) where appropriate. Repeated measures mixed models were used for the urine pH and sUA analysis in the per-protocol (PP) population. A Bonferroni correction was used among 3 treatment comparisons at an alpha level of 0.016. The comparison of response rates based on the distribution of urine pH and sUA levels was calculated by chi-square test among the three groups. The changes in UACR were analyzed by a generalized estimation equation model with baseline values as covariates. Comparisons before and after treatment within the group were made using paired *T* or rank sum tests. Gout flares were compared by negative binomial regression following the intent-to-treat (ITT: All randomized patients who received ≥ 1 dose of randomized study medication were included in the ITT population) principle, the same population also for adverse events assessments. The baseline and 12 week’s follow-up analyses were performed by the PP principle. SPSS 25.0 (IBM SPSS, Chicago, IL, USA) and R version 4.2.1 (https://www.r-project.org) were used for statistical analysis. All tests were two-sided, and *P* < 0.05 was regarded as statistically significant.

## Results

### Patients and baseline characteristics

**Fig. 1:**
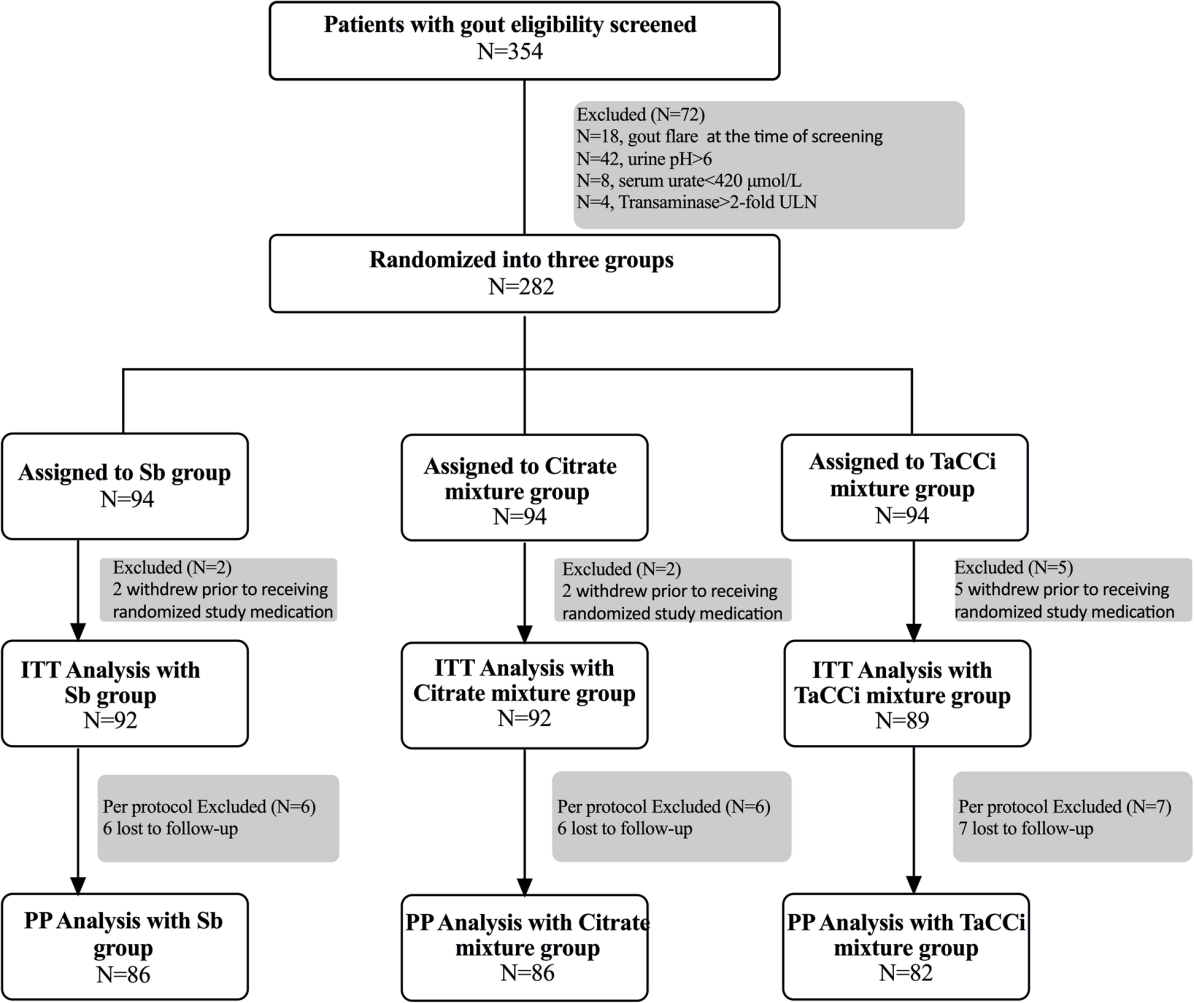
Flow of study participants. Totally 354 participants were screened, and then 282 eligible patients were enrolled and randomized into sodium bicarbonate, citrate mixture, and TaCCi mixture groups. Nine participants withdrew before receiving at least 1 dose of treatment medication and were excluded from ITT analysis; furthermore, 19 participants were excluded from PP analysis for loss to follow-up. Sb, sodium bicarbonate; TaCCi mixture, tart cherry supplementary citrate mixture, ITT, intention to treat; PP, per protocol

As shown in Fig. 1, a total of 354 patients were screened; 72 patients were excluded for the following reasons: gout flare (*n* = 18), fasting urine pH > 6 (*n* = 42), sUA < 420 μmol/L (*n* = 8), transaminase > two folds of ULN (*n* = 4); 282 participants were randomized into the sodium bicarbonate, citrate mixture, or TaCCi mixture group at 1:1:1 ratio; and finally, 254 participants completed the study (86 in sodium bicarbonate group, 86 in citrate mixture group, and 82 in TaCCi mixture group). The first study visit was September 2021 and the final study visit was June 2022. Of the 282 participants, 9 withdrew before receiving at least 1 dose of treatment medication, the remaining 273 participants were included in the ITT analyses, and 254 participants who completed the study were included in the PP analyses (Fig. 1).

At baseline, the demographic and clinical features were comparable across these three groups. As shown in Table 1, the median (interquartile range) baseline urine pH levels were 5.56 (5.32–5.77) in the sodium bicarbonate group, 5.42 (5.25–5.88) in the citrate mixture group and 5.51 (5.34–5.72) in the TaCCi mixture group. The mean ± S.D of baseline sUA levels in these 3 groups were 538 ± 68.3 μmol/L, 533 ± 59.3 μmol/L, and 524 ± 68.1 μmol/L respectively (Table 1).

**Table 1:** Baseline demographics and clinical features of gout patients

	**Sodium bicarbonate (*N* = 86)**	**Citrate mixture (*N* = 86)**	**TaCCi mixture (*N* = 82)**
**Age, years**	42.0 (35.0–55.5)	45.5 (35.0–55.5)	47.0 (34.8–57.0)
**BMI, kg/m**^**2**^	26.8 (24.8–29.0)	27.2 (25.0–28.9)	27.4 (25.3–29.1)
**Systolic blood pressure, mmHg**	135 ± 15.2	135 ± 15.5	140 ± 17.2
**Diastolic blood pressure, mmHg**	86.8 ± 10.9	87.9 ± 10.8	89.1 ± 11.2
**Nephrolithiasis, *n* (%)**	9 (10.5%)	6 (6.98%)	3 (3.66%)
**Renal cyst**	24 (27.9%)	23 (26.7%)	23 (28.0%)
**Tophi, *n* (%)**	8 (9.30%)	4 (4.65%)	3 (3.66%)
**Smoking, *n* (%)**	42 (48.8%)	31 (36.0%)	38 (46.3%)
**Drinking, *n* (%)**	51 (59.3%)	33 (38.4%)	41 (50.0%)
**Blood laboratory parameters**			
Serum urate, μmoI/L	538 ± 68.3	533 ± 59.3	524 ± 68.1
Triglyceride, mmol/L	1.62 (1.16–2.81)	1.76 (1.27–2.42)	1.76 (1.21–2.56)
Total Cholesterol, mmol/L	5.28 ± 0.98	5.07 ± 0.99	5.28 ± 1.03
Fasting blood glucose, mmol/L	5.83 (5.61–6.30)	5.89 (5.53–6.39)	5.87 (5.57–6.18)
HOMA-IR	3.01 (2.36–4.70)	4.19 (2.26–6.21)	3.48 (2.34–5.57)
ALT, U/L	24.0 (18.0–38.0)	24.0 (18.0–40.0)	26.0 (19.0–37.3)
AST, U/L	21.0 (18.0–24.3)	21.0 (18.0–27.0)	21.5 (18.0–25.0)
Blood urea nitrogen, mmol/L	4.95 (4.28–5.90)	4.75 (4.18–5.90)	5.00 (4.30–5.90)
Serum creatinine, μmoI/L	81.5 (73.8–92.3)	84.0 (76.0–90.0)	84.0 (78.0–93.0)
eGFR, mL/(min·1.73 m^2^)	94.6 ± 16.7	93.5 ± 16.1	91.5 ± 15.2
Serum potassium, mmol/L	4.30 (4.10–4.53)	4.40 (4.10–4.50)	4.30 (4.20–4.50)
Serum sodium, mmol/L	143 (142–144)	142 (142–144)	142 (141–143)
Serum chlorine, mmol/L	102 (100–104)	102 (100–103)	103 (102–104)
CRP, mg/L	3.30 (2.40–4.50)	3.60 (2.65–4.15)	3.50 (2.40–4.20)
**Urine parameters**			
pH	5.56 (5.32–5.77)	5.42 (5.25–5.88)	5.51 (5.34–5.72)
Proteinuria, *n* (%)	8 (9.30%)	9 (10.5%)	5 (6.10%)
Hemoglobinuria, *n* (%)	3 (3.49%)	4 (4.65%)	3 (3.66%)
UACR, mg/g	3.01 (0.56–11.2)	3.29 (0.44–10.3)	7.42 (0.72–11.9)

Data were shown as mean ± standard derivation (S.D), median (interquartile range), or number (percentage)

*BMI* body mass index, *HOMA-IR* homeostasis model assessment-insulin resistance, *ALT* alanine aminotransferase, *AST* aspartate aminotransferase, *eGFR* estimated glomerular filtration rate, *CRP* C-reactive protein, *UACR* 24-h urinary albumin- creatinine ratio, *TaCCi* mixture, tart cherry supplementary citrate mixture

### Primary outcomes

**Fig. 2:**
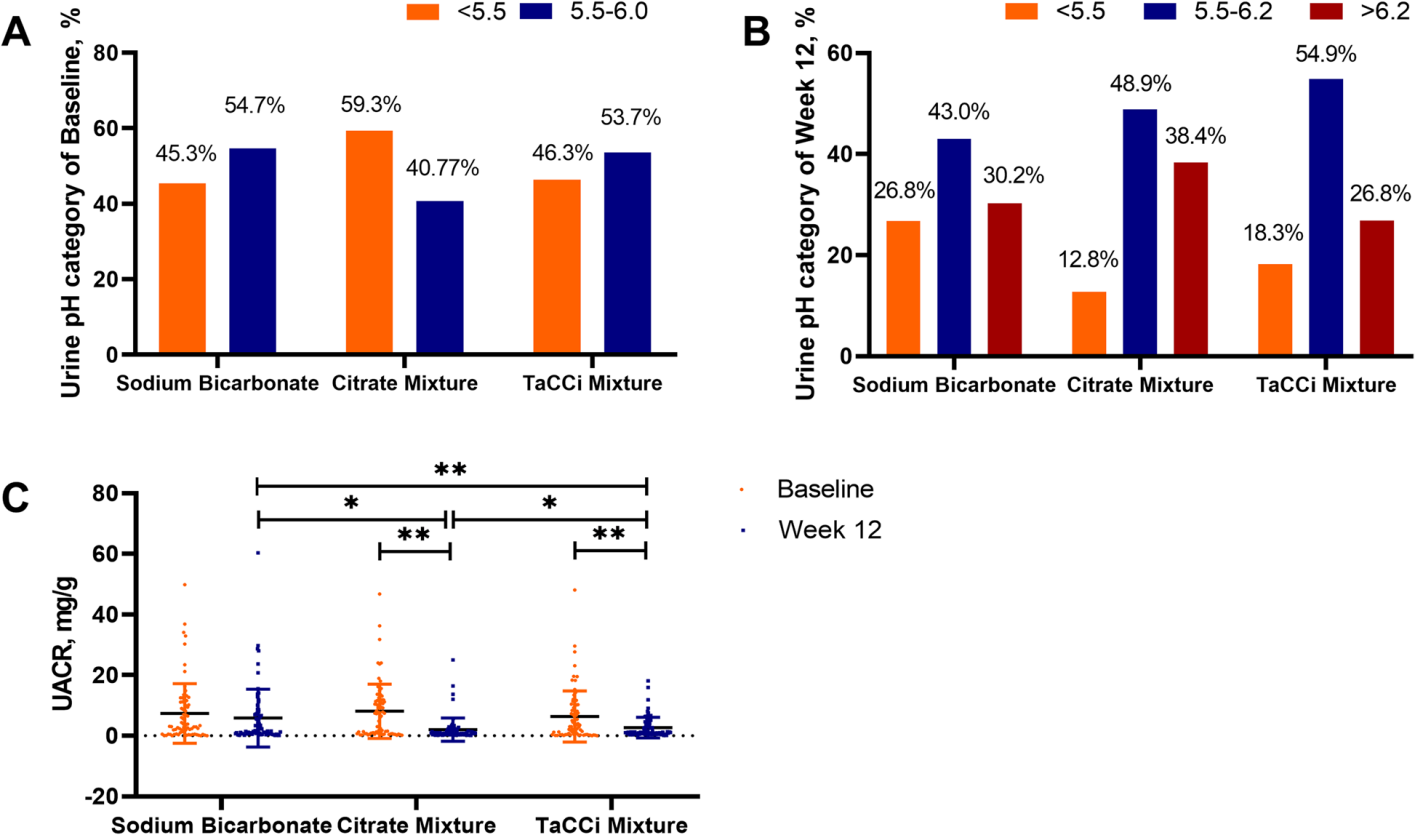
Urine pH categories and UACR changes. **A** Proportion of patients with pH < 5.5 or ≥ 5.5 at baseline. **B** Proportion of patients with pH < 5.5, 5.5–6.2, or > 6.2 at week 12. **C** UACR at baseline and week 12. TaCCi mixture, tart cherry supplementary citrate mixture group; UACR, 24-h urinary albumin-creatinine ratio

The urine pH increased in all groups at week 4 and remained stable until week 12, median (interquartile range) 5.56 (5.32–5.77) to 5.82 (5.47–6.40) in the sodium bicarbonate group, 5.42 (5.25–5.88) to 6.02 (5.67–6.65) in the citrate mixture group, and 5.51 (5.34–5.72) to 5.88 (5.55–6.28) in the TaCCi mixture group, respectively, with no significant difference observed between groups in pH level, nor in the proportion when sub-grouped with pH (< 5.5, 5.5–6.2, and > 6.2) (Table 2, Fig. 2A, B), and age did not change the treatment response of the 3 groups (Table S1).

**Table 2:** Effect of treatment on renal parameters and metabolic syndrome components in the per-protocol set

	**Baseline**	**Week 4**	**Week 8**	**Week 12**
**Urine pH, median (IQR)**				
**Sodium bicarbonate**	5.56 (5.32–5.77)	5.76 (5.43–6.35)	5.80 (5.46–6.10)	5.82 (5.47–6.40)
**Citrate mixture**	5.42 (5.25–5.88)	6.04 (5.54–6.49)	5.89 (5.51–6.38)	6.02 (5.67–6.65)
**TaCCi mixture**	5.51 (5.34–5.72)	5.79 (5.46–6.25)	5.74 (5.50–6.32)	5.88 (5.55–6.28)
**Serum urate, mean** ± **S.D, μmol/L**				
**Sodium bicarbonate**	538 ± 68.3	380 ± 71.9*	358 ± 61.5*	370 ± 82.3*
**Citrate mixture**	533 ± 59.3	371 ± 67.1*	350 ± 56.4*	360 ± 64.6*
**TaCCi mixture**	524 ± 68.1	369 ± 68.7*	357 ± 60.2*	360 ± 59.0*
**Serum urate < 360 μmol/L, patients (%)**				
**Sodium bicarbonate**	-	37 (43.0%)	45 (52.3%)	46 (53.5%)
**Citrate mixture**	-	39 (45.4%)	53 (61.6%)	49 (57.0%)
**TaCCi mixture**	-	40 (48.8%)	45 (54.9%)	45 (54.9%)
**Serum urate < 300 μmol/L, patients (%)**				
**Sodium bicarbonate**	-	6 (7.00%)	12 (14.0%)	14 (16.3%)
**Citrate mixture**	-	10 (11.6%)	10 (11.6%)	13 (15.1%)
**TaCCi mixture**	-	9 (11.0%)	12 (14.6%)	14 (17.1%)
**Body mass index, median (IQR), kg/m**^**2**^				
**Sodium bicarbonate**	26.8 (24.8–29.0)	27.0 (24.8–28.8)	27.0 (24.5–28.7)	27.2 (24.8–28.9)*
**Citrate mixture**	27.2 (25.0–28.9)	27.2 (25.3–28.9)	27.3 (25.0–28.9)	27.3 (25.1–28.9)
**TaCCi mixture**	27.4 (25.3–29.1)	27.3 (25.4–29.2)	27.4 (25.6–29.0)	27.4 (25.5–29.1)
**Systolic blood pressure, median (IQR), mmHg**				
**Sodium bicarbonate**	135 (124–148)	135 (124–148)	138 (126–149)*	137 (130–151)*
**Citrate mixture**	135 (122–144)	137 (124–145)	137 (126–147)	139 (128–147)
**TaCCi mixture**	136 (128–149)	137 (126–147)	136 (127–145)	133 (123–145)*
**Diastolic blood pressure, mean** ± **S.D, mmHg**				
**Sodium bicarbonate**	86.8 ± 10.9	88.4 ± 10.2	89.2 ± 10.1	88.9 ± 10.3
**Citrate mixture**	87.9 ± 10.8	88.1 ± 10.6	88.0 ± 10.5	87.6 ± 10.4
**TaCCi mixture**	89.1 ± 11.2	87.2 ± 11.2	87.7 ± 10.4	85.9 ± 10.5*
**Fasting blood glucose, median (IQR), mmol/L**				
**Sodium bicarbonate**	5.83 (5.61–6.30)	5.65 (5.32–6.19)	5.69 (5.43–6.07)*	5.86 (5.49–6.23)
**Citrate mixture**	5.89 (5.53–6.39)	5.79 (5.39–6.25)*	5.63 (5.42–6.07)*	5.84 (5.44–6.20)
**TaCCi mixture**	5.87 (5.57–6.18)	5.69 (5.33–6.01)*	5.63 (5.28–5.97)*	5.67 (5.29–5.90)*
**Homeostasis model assessment-insulin resistance, median (IQR)**				
**Sodium bicarbonate**	3.01 (2.36–4.70)	2.68 (1.99–3.97)	2.73 (2.13–3.66)	2.76 (1.83–4.15)
**Citrate mixture**	4.19 (2.26–6.21)	3.22 (2.37–5.12)	3.79 (2.57–5.44)	3.26 (2.31–5.33)
**TaCCi mixture**	3.48 (2.34–5.57)	3.06 (1.92–4.63)*	2.74 (2.01–4.79)*	3.00 (2.04–5.21)*
**Triglyceride, median (IQR), mmol/L**				
**Sodium bicarbonate**	1.62 (1.16–2.81)	1.84 (1.31–2.69)	1.77 (1.30–2.50)	1.60 (1.21–2.50)
**Citrate mixture**	1.76 (1.27–2.42)	1.68 (1.17–2.40)	1.61 (1.11–2.27)	1.53 (1.23–2.32)
**TaCCi mixture**	1.76 (1.21–2.56)	1.82 (1.23–2.34)	1.97 (1.34–2.61)	1.77 (1.31–2.35)
**Total cholesterol, median (IQR), mmol/L**				
**Sodium bicarbonate**	5.23 (4.73–5.81)	5.34 (4.57–5.75)	5.21 (4.66–5.85)	5.28 (4.72–5.99)
**Citrate mixture**	5.07 (4.37–5.73)	4.86 (4.38–5.67)	4.96 (4.27–5.72)	5.00 (4.29–5.80)
**TaCCi mixture**	5.24 (4.71–5.94)	5.24 (4.56–5.84)	5.17 (4.59–5.81)	5.14 (4.37–5.63)*
**Blood urea nitrogen, median (IQR), mmol/L**				
**Sodium bicarbonate**	4.95 (4.28–5.90)	5.00 (4.30–6.00)	5.10 (4.50–5.80)	5.10 (4.20–5.90)
**Citrate mixture**	4.75 (4.18–5.90)	5.25 (4.60–6.28)	5.70 (4.60–6.25)*	5.40 (4.58–6.23)
**TaCCi mixture**	5.00 (4.30–5.90)	5.00 (4.45–5.90)	5.30 (4.63–5.90)	5.00 (4.30–6.00)
**Serum creatinine, median (IQR), μmoI/L**				
**Sodium bicarbonate**	81.5 (73.8–92.3)	82.0 (75.8–95.0)	81.0 (74.0–91.0)	83.5 (77.0–92.3)
**Citrate mixture**	84.0 (76.0–90.0)	85.0 (76.0–92.0)	83.0 (75.0–93.0)	85.5 (79.0–93.3)
**TaCCi mixture**	84.0 (78.0–93.0)	82.0 (76.0–93.0)	81.0 (73.3–89.8)*	84.0 (77.0–91.0)
**Estimated glomerular filtration rate, mean** ± **S.D, mL/min/1.73 m**^**2**^				
**Sodium bicarbonate**	94.6 ± 16.7	94.3 ± 18.2	95.0 ± 17.1	92.7 ± 17.0
**Citrate mixture**	93.5 ± 16.1	93.0 ± 15.8	94.5 ± 16.9	90.8 ± 16.1
**TaCCi mixture**	91.5 ± 15.2	93.1 ± 16.4	97.1 ± 17.1*	92.4 ± 15.0
**C-reactive protein, median (IQR), mg/L**				
**Sodium bicarbonate**	3.30 (2.40–4.50)	-	-	3.30 (2.40–5.90)
**Citrate mixture**	3.60 (2.65–4.15)	-	-	3.25 (2.90–4.53)
**TaCCi mixture**	3.50 (2.40–4.20)	-	-	2.00 (2.00–2.80)**^##††^
**Urinary albumin-creatinine ratio, median (IQR), mg/g**				
**Sodium bicarbonate**	3.01 (0.56–11.2)	-	-	1.72 (0.73–7.08)
**Citrate mixture**	3.29 (0.44–10.3)	-	-	1.17 (0.71–4.14)**^#^
**TaCCi mixture**	7.42 (0.72–11.9)	-	-	0.89 (0.40–1.82)**^##†^
**Patients with hemoglobinuria (*n* (%))**				
**Sodium bicarbonate**	3 (3.49%)	2 (2.33%)	1 (1.16%)	1 (1.16%)
**Citrate mixture**	4 (4.65%)	3 (3.49%)	2 (2.33%)	3 (3.49%)
**TaCCi mixture**	3 (3.66%)	2 (2.44%)	1 (1.22%)	1 (1.22%)

Data were shown as mean ± standard derivation (S.D) or median (interquartile range, IQR)

*TaCCi mixture*, tart cherry supplementary citrate mixture

*compared to baseline *p* < 0.05, ** compared to baseline *p* < 0.01; ^#^compared to sodium bicarbonate *p* < 0.05, ^##^compared to sodium bicarbonate *p* < 0.01; † compared to citrate mixture *p* < 0.05; †† compared to citrate mixture *p* < 0.01

The sUA level decreased significantly in all groups (mean ± S.D) 538 ± 68.3 to 370 ± 82.3 μmol/L in the sodium bicarbonate group, 533 ± 59.3 to 360 ± 64.6 μmol/L in the citrate mixture group, and 524 ± 68.1 to 360 ± 59.0 μmol/L in the TaCCi mixture group. There were no significant differences between groups in sUA level, nor in the proportion achieving sUA targets (sUA < 360 or < 300 μmol/L) at each visit (Table 2), and age didn’t change the treatment response of the 3 groups (Table S1).

### Gout-related outcomes

The incidence of gout flare in the intent-to-treat population is shown in Table S2; the proportion of patients with gout flares at weeks 4, 8, and 12 was 25 (27.2%), 28 (30.4%), and 19 (20.7%) in the sodium bicarbonate group; 16 (17.4%), 22 (23.9%), and 15 (16.3%) in the citrate mixture group; and 18 (20.2%), 14 (15.7%), and 14 (15.7%) in the TaCCi mixture group. Participants in the citrate mixture group and the TaCCi mixture group experienced fewer gout flares than participants in the sodium bicarbonate group during the study (0.58 ± 0.87 and 0.56 ± 0.88 per participant versus 0.94 ± 1.45 per participant, p = 0.033 and 0.024, respectively), with no difference between the citrate mixture group and the TaCCi mixture group (Table S2). For CRP there was no significant difference between groups at baseline, while it was lower in TaCCi mixture at week 12 when compared to the other two groups (*p* < 0.01) (Table 2).

The DECT detected urate volume (Ln transformed) decreased significantly from baseline in all groups, whereas no significant between-group difference was observed at baseline or at week 12 (Fig. S1).

### Renal and metabolic outcomes

No significant changes in eGFR were observed in the groups over time, except for an increase at the week 8 time point in the TaCCi mixture group (Table 2). The UACR improved from 3.01 (0.56–11.2) mg/g to 1.72 (0.73–7.08) mg/g in the sodium bicarbonate group (*p* = 0.253), from 3.29 (0.44–10.3) mg/g to 1.17 (0.71–4.14) mg/g in the citrate mixture group (*p* = 0.007) and from 7.42 (0.72–11.9) mg/g at baseline to 0.89 (0.40–1.82) mg/g at week 12 in the TaCCi mixture group (*p* < 0.001) (Table 2). Notably, significant differences in UACR were observed between groups at week 12, of which the mean difference between the sodium bicarbonate group and the TaCCi mixture group was − 4.40 mg/g (95% CI, − 6.87 to − 1.94, *p* < 0.001)), between the citrate mixture group and the TaCCi mixture group was − 1.51 mg/g (95% CI, − 2.94 to − 0.085, *p* = 0.034) and between the sodium bicarbonate group and the citrate mixture group was − 2.89 mg/g (95%CI, − 5.46 to –0.81, *p* = 0.021) (Fig. 2C).

As for MetS components, in the TaCCi mixture group, the SBP, DBP, and TC levels decreased significantly from baseline to week 12, and the FBG level as well as HOMA-IR decreased significantly from baseline at all visits (*p* < 0.05). In the sodium bicarbonate group, SBP and BMI increased from baseline to week 12 (*p* < 0.05) (Table 2). However, no significant between-group differences were observed at any visit (Table 2).

### Adverse events

The serum electrolytes, Na^+^, K^+^, and Cl^−^, were similar between groups and over time (Fig. S2A–C). There was no significant difference regarding adverse events across the three groups: the incidence of newly-onset hemoglobinuria was 4 (4.35%), 2 (2.17%), and 3 (3.37%); newly-onset nephrolithiasis was 1 (1.09%), 2 (2.17%) and 1 (1.12%); newly-onset renal cyst was 6 (6.98%), 4 (4.65%), and 2 (2.44%); newly-onset hypertension was 3 (3.26%), 3 (3.26%), and 1 (1.12%) in sodium bicarbonate, citrate mixture, and TaCCi mixture group, respectively (Table 3). All three treatments were well tolerated, and no other adverse events that might lead to treatment interruption or hospitalization occurred during this study.

**Table 3:** Incidence of adverse events (intention to treat analysis)

	Sodium bicarbonate (*N* = 92)	Citrate mixture (*N* = 92)	TaCCi mixture (*N* = 89)	*P*
	Patients (%)	Patients (%)	Patients (%)	
Newly-onset hemoglobinuria	4 (4.35%)	2 (2.17%)	3 (3.37%)	0.710
Newly-onset nephrolithiasis	1 (1.09%)	2 (2.17%)	1 (1.12%)	0.710
Newly-onset renal cyst	6 (6.98%)	4 (4.65%)	2 (2.44%)	0.383
Newly-onset hypertension	3 (3.26%)	3 (3.26%)	1 (1.12%)	0.785
Skin allergy	0 (0.00%)	1 (1.09%)	0 (0.00%)	0.578
ALT				
1–2 × ULN	14 (15.2%)	12 (13.0%)	16 (18.0%)	0.373
2–3 × ULN	1 (1.09%)	0 (0.00%)	2 (2.25%)	0.654
> 3 × ULN	0 (0.00%)	1 (1.09%)	0 (0.00%)	0.350
AST				0.373
1–2 × ULN	4 (4.35%)	7 (7.61%)	10 (11.2%)	0.220
2–3 × ULN	0 (0.00%)	0 (0.00%)	0 (0.00%)	1.00
> 3 × ULN	1 (1.09%)	0 (0.00%)	0 (0.00%)	0.373

*ALT* alanine aminotransferase, *AST* aspartate aminotransferase, *ULN* upper limit of normal, *TaCCi mixture* tart cherry supplementary citrate mixture

## Discussion

The aim of this study was to investigate the efficacy and safety of a tart cherry supplementary citrate (TaCCi) mixture on urine pH, sUA, and gout flares in gout patients initiating ULT with XOI febuxostat. The hypothesis was that tart cherry supplement could ameliorate acidic urine by improving MetS components. Our findings indicate that the TaCCi mixture had similar efficacy and safety to sodium bicarbonate or citrate mixture for urine alkalization. However, the TaCCi mixture group demonstrated greater improvement in UACR and CRP compared to the other two groups, and fewer gout flares than the sodium bicarbonate group. No between-group differences were observed for MetS components over the 12-week study period.

Low urine pH is a major risk factor for renal system uric acid crystallization formation and stone development [29]. However, a prior study found that urine alkalization with sodium bicarbonate for three months did not retard the progress of kidney damage as assessed by eGFR and hematuria in patients with gout, though citrate mixture lowered hematuria at comparable urine pH levels [23]. In our study, both the TaCCi mixture and citrate mixture significantly reduced the UACR after 12 weeks, with UACR being lower in the TaCCi mixture group than in the citrate mixture or the sodium bicarbonate groups at week 12. UACR is a sensitive and reliable index for glomeruli injury and a key criterion in CKD classification as recommended by the KDIGO guidelines [30]. The improvement of UACR may partly reflect citrate-induced resolution of uric acid crystal in renal tubules relieving pressure in the Bowman's capsule of glomerulus, as suggested by our prior study [23]. The greater reductions of UACR in the TaCCi mixture group align with anthocyanins from tart cherry lowering UACR in high-fat diet mice [31]. Anthocyanins and metabolites possess potent anti-oxidative and anti-inflammatory properties, and may attenuate renal injury in mice through reduced oxidative stress and protected mitochondrial function [32]. Uric acid crystals can induce renal inflammatory, proliferative and maladaptive changes, manifesting as renal cysts, nephrolithiasis, and eventual CKD, while soluble urate may similarly impact glomeruli in a manner unaffected by urine pH [33]. Oxidative stress and activation of the NLRP3 inflammasome in kidney cells correlate with these changes [33]. Taken together, the TaCCi mixture may provide extra renal protection, though the mechanism requires further study.

Observational studies have suggested that tart cherry intake may reduce gout flare frequency [17, 34]. Additionally, in vitro studies showed tart cherry inhibits IL-1β secretion, closely linked to gout flare initiation [34]. The urate-lowering effects of tart cherry are controversial, with variable results reported [16, 17, 34, 35]. The current data did not find greater serum urate-lowering among study participants all initiating febuxostat during the study period. In addition, the incidence of gout flare was lower in both the TaCCi mixture and the citrate mixture groups versus the sodium bicarbonate group. Decreased gout flares with the citrate mixture agree with prior work [23]. Citrate participates in the tricarboxylic acid cycle and other multiple metabolic pathways [36]. During inflammation, citrate withdraws from the Krebs cycle and exports to the cytosol, where it is cleaved and exerts anti-inflammatory function [36]. Lower gout flare incidence with citrate and TaCCi mixture may relate to their citrate component. CRP levels were similar at baseline, but decreased more in the TaCCi mixture group compared with the other two groups at week 12. CRP elevation reflects inflammation from potentially diverse sources of gout. These data suggested that tart cherry supplement lowered systemic inflammation in gout patients at 12 weeks, although gout flares were similar between the TaCCi and the citrate mixture groups.

The study had several limitations. Firstly, it was conducted at a single clinical center and was open label. Secondly, only male patients with eGFR > 60 ml/min/1.73m^2^ were enrolled. Thirdly, as a short-term study, long-term efficacy, safety, and impacts on other conditions of the mixtures remain unclear. Thus, a double-blind, placebo-controlled study of varied patients over longer follow-up is needed to better evaluate the role of urine alkalinization in gout management and confirm these results.

## Conclusions

This study demonstrated that tart cherry supplementary citrate mixture has similar efficacy on urine pH in gout patients initiating XOI therapy when compared to a citrate mixture and sodium bicarbonate. The tart cherry mixture also showed similar safety. However, the tart cherry mixture resulted in greater improvements in UACR and CRP levels, which suggests that tart cherry supplements might offer extra therapeutic value beyond just urine alkalization.

### Supplementary Information

**Additional file 1: Table S1.** Age related treatment response of the three groups:Participants in the three treatment arms were grouped into > 40 years old or ≤ 40 years old subgroup. Those main outcomes were compared between participants of each subgroup within and between/among these three treatment groups, including ΔUACR, ΔSU, ΔpH, pH ≥ 6.2 at week12 and SU < 360 μmol/L at week 12 in vs in three treatment groups, and no significant difference was observed between subgroups. **Table S2.** Gout flares in the intent-to-treat set. **Fig. S1.** DECT urate volumes. Significant differences (*p* < 0.05) were observed in Sodium bicarbonate, Citrate mixture and TaCCi mixture group between baseline and week 12, while no significant difference was observed between groups. TaCCi Mixture: Tart Cherry supplementary Citrate Mixture. **Fig. S2.** Serum electrolytes levels during follow up. No significant difference was observed between baseline and week 12 in Sodium bicarbonate, Citrate mixture and TaCCi mixture group. A: Serum potassium,B: serum sodium and C: serum chlorine levels.

## Data Availability

The datasets used and/or analyzed during the current study are available from the corresponding author on reasonable request.

## Abbreviations

MetS: Metabolic syndrome
CKD: Chronic kidney disease
TC: Total cholesterol
LDL-C: Low-density lipoprotein cholesterol
HDL-C: High-density lipoprotein cholesterol
FBG: Fasting blood glucose
sUA: Serum urate
ACR: American College of Rheumatology
ULT: Urate-lowing therapy
XOI: Xanthine oxidase inhibitors
UACR: Twenty-four-hour urinary albumin/creatinine ratio
TaCCi: Tart cherry supplementary citrate
eGFR: Estimated glomerular filtration rate
ULN: Upper limit of normal
CRP: C-reactive protein
TG: Triglycerides
HOMA-IR: Homeostasis model assessment of insulin resistance
BUN: Blood urea nitrogen
CREA: Serum creatinine
BP: Blood pressure
BMI: Body mass index
DECT: Dual-energy CT
MSU: Monosodium urate
S.D: Standard derivation
PP: Per protocol
ITT: Intent-to-treat
KDIGO: Kidney Disease: Improving Global Outcomes
NLRP3: NOD-like receptor thermal protein domain associated protein 3
