# Supplementary material for: Efficacy and safety of tart cherry supplementary citrate mixture on gout patients: a prospective, randomized, controlled study
Source: Arthritis Res Ther. 2023 Sep 7;25:164. doi: 10.1186/s13075-023-03152-1 (PMC10483724; doi:10.1186/s13075-023-03152-1)
Supplement: Supplementary file 1 — Additional file 1: Table S1. Age related treatment response of the three groups:Participants in the three treatment arms were grouped into > 40 years old or ≤ 40 years old subgroup. Those main outcomes were compared between participants of each subgroup within and between/among these three treatment groups, including ΔUACR, ΔSU, ΔpH, pH ≥ 6.2 at week12 and SU < 360 μmol/L at week 12 in vs in three treatment groups, and no significant difference was observed between subgroups. Table S2. Gout flares in the intent-to-treat set. Fig. S1. DECT urate volumes. Significant differences (p < 0.05) were observed in Sodium bicarbonate, Citrate mixture and TaCCi mixture group between baseline and week 12, while no significant difference was observed between groups. TaCCi Mixture: Tart Cherry supplementary Citrate Mixture. Fig. S2. Serum electrolytes levels during follow up. No significant difference was observed between baseline and week 12 in Sodium bicarbonate, Citrate mixture and TaCCi mixture group. A: Serum potassium,B: serum sodium and C: serum chlorine levels. [file 13075_2023_3152_MOESM1_ESM.zip › Supplementary file.docx]

**Efficacy and Safety of Tart Cherry Supplementary Citrate Mixture on Gout Patients: A Prospective, Randomized, Controlled Study**

**Table S1. Age related treatment response of the three groups:**Participants in the three treatment arms were grouped into ＞40 years old or ≤40 years old subgroup. Those main outcomes were compared between participants of each subgroup within and between/among these three treatment groups, including ΔUACR, ΔSU, ΔpH, pH≥6.2 at week12 and SU<360μmol/L at week 12 in vs in three treatment groups, and no significant difference was observed between subgroups

**Table S2.** **Gout flares in the intent-to-treat set**

**Fig. S1. DECT urate volumes**. Significant differences (p<0.05) were observed in Sodium bicarbonate, Citrate mixture and TaCCi mixture group between baseline and week 12, while no significant difference was observed between groups. TaCCi Mixture: Tart Cherry supplementary Citrate Mixture.

**Fig. S2. Serum electrolytes levels during follow up.** No significant difference was observed between baseline and week 12 in Sodium bicarbonate, Citrate mixture and TaCCi mixture group. **A:** Serum potassium,B: serum sodium and C: serum chlorine levels.

**Table S1. Age related treatment response of the three groups**

|  | Sodium bicarbonate | |  | Citrate mixture | |  | TaCCi Mixture | |
| --- | --- | --- | --- | --- | --- | --- | --- | --- |
|  | ≤40 years | ＞40 years |  | ≤40 years | ＞40 years |  | ≤40 years | ＞40 years |
| ΔSU (μmol/L) | -180±124 | -157±111 |  | -192±79 | -161±65 |  | -171±56 | -160±71 |
| ΔpH | 0.31±0.36 | 0.32±0.64 |  | 0.60±0.65 | 0.59±0.62 |  | 0.42±0.48 | 0.38±0.46 |
| pH≥6.2 at week12 | 13/40(32.5%) | 12/46(26.1%) |  | 14/35(40.0%) | 18/51(35.3%) |  | 9/31(29.0%) | 14/51(27.5%) |
| SU<360 μmol/L at week 12 | 20/40(50.0%) | 27/46(58.7%) |  | 18/35(51.4%) | 29/51(56.7%) |  | 16/31(51.6%) | 29/51(56.95%) |

Δ: week12-baseline; *p<0.05; SU: serum urate; UACR: urine albumin/creatinine ratio.

**Table S2.** **Gout flares in the intent-to-treat set**

|  | | **Sodium bicarbonate**  **(n=92)** | **Citrate mixture (n=92)** | **TaCCi mixture (n=89)** | **P value** |
| --- | --- | --- | --- | --- | --- |
| **0-4 week** | | 25 (27.2%) | 16 (17.4%) | 18 (20.2%) | 0.253 |
| **4-8 week** | | 28 (30.4%) | 22 (23.9%) | 14 (15.7%) | 0.065 |
| **8-12 week** | | 19 (20.7%) | 15 (16.3%) | 14 (15.7%) | 0.634 |
| **Flares over the 12-week period** |  | | | | |
| Once | | 42 (45.7%) | 36 (39.1%) | 35 (39.3%) | 0.595 |
| ≥Twice | | 21 (22.8%) | 13 (14.1%) | 11 (12.4%) | 0.125 |
| **Average number of flares over the 12-week period** | | 0.94±1.45 | 0.58±0.87  p=0.033* | 0.56±0.88  p=0.024* | <0.05 |

**TaCCi mixture：**Tart Cherry supplementary Citrate Mixture. * versus Sodium bicarbonate group p<0.05.
